# Case Report: A successful multidisciplinary approach to doxorubicin extravasation from a PICC-port in a patient with breast cancer

**DOI:** 10.3389/fonc.2025.1534112

**Published:** 2025-07-18

**Authors:** Concetta Calabrò, Serena Iacovelli, Giuseppe De Palma, Giuseppe Carravetta, Domenica Garofalo, Francesco Giotta, Agnese Latorre, Patrizia Nardulli, Cosmo Maurizio Ressa, Elsa Vitale, Valerio De Santis, Giovanni Mastrandrea

**Affiliations:** ^1^ Pharmacy Unit, IRCCS Istituto Tumori “Giovanni Paolo II” Bari, Bari, Italy; ^2^ Institutional BioBank, Experimental Oncology and Biobank Management Unit, IRCCS Istituto Tumori “Giovanni Paolo II” Bari, Bari, Italy; ^3^ Anaesthesia, Resuscitation and Postoperative Intensive Care Unit, IRCCS Istituto Tumori “Giovanni Paolo II” Bari, Bari, Italy; ^4^ Medical Oncology Unit, IRCCS Istituto Tumori “Giovanni Paolo II” Bari, Bari, Italy; ^5^ Plastic and Reconstructive Surgery Unit, IRCCS Istituto Tumori “Giovanni Paolo II” Bari, Bari, Italy; ^6^ Scientific Directorate, IRCCS Istituto Tumori “Giovanni Paolo II” Bari, Bari, Italy

**Keywords:** breast cancer, intravenous chemotherapy drugs, doxorubicin extravasation, multidisciplinary intervention, long-term central venous access, PICC-PORT, necrosis

## Abstract

**Background:**

Infusion of chemotherapy drugs through central venous catheters in the bloodstream facilitates direct access to disseminated cancer sites to interrupt the growth and/or spread of abnormal cells. To represent the significance of a rapid, multidisciplinary intervention codified by a hospital-adopted procedure for the treatment of this specific type of extravasation.

**Methods:**

A case of a 63-year-old female patient with no comorbidities but overweight who was admitted to our hospital in 2023 was discussed. The oncologist requested the placement of a long-term central venous access for chemotherapy, expected to last at least 5–6 months. This case report describes a massive anthracycline extravasation through a PICC-port. Such a serious complication requires not only the prompt administration of dexrazoxane but, more importantly, a multidisciplinary approach. Without comprehensive and timely intervention, the patient would have likely lost the upper limb.

**Clinical implications:**

Following the surgical and pharmacological treatment, the patient achieved a restoration of normal limb function, thus resuming all regular activities. This outcome was made possible primarily due to the timely and professional intervention of the multidisciplinary team, which minimized the severe complications that doxorubicin extravasation can cause. Tunneling of the catheter, which moves the extravasation site (port pocket) away from the venipuncture site, is equally important. Another noteworthy element is the resumption of chemotherapy treatment, which might have been interrupted due to the severe complication resulting from the extravasation.

## 1 Introduction

Infusion of intravenous chemotherapy drugs in the bloodstream facilitates direct access to disseminated cancer sites to interrupt the growth and/or spread of abnormal cells. To reduce complication and to facilitate this infusion, the most preferred options are central vascular access devices, including peripherally inserted central catheters (PICCs) and totally implantable vascular access devices (TIVADs) with chest insertion (chest port) or peripheral insertion (arm port or PICC port) (1, 2). Also, in economic studies published in the last 5 years, the TIVADs are starting to be considered more cost-effective than CVCs and PICCs in breast cancer chemotherapy patients (3–7).

Moreover, the cosmetic and psychological advantage has led to a more frequent use of peripherally inserted TIVADs in breast cancer: the additional scar in a hidden area of the body, the non-need to uncover and use the chest, and the non-need to carry out weekly PICC medications justify breast cancer patients preferring these devices (1). Weekly maintenance includes the use of needle-free connectors, sutureless securement devices, and transparent semipermeable dressings. In addition, PICCs require a weekly flush to ensure catheter patency and prevent occlusions (8).

In order to reduce the incidence of injury during the peripheral insertion of TIVADs, various studies close to the Italian healthcare system recommend using ultrasound guidance in real time, maximum barrier protection, the micro-Seldinger technique for the venipuncture in the proximal third of the upper arm, close to the axilla, and the tunneling up to a pocket for the port chamber located in the “green zone” of the zone insertion method (ZIM) used for PICCs (1–7, 9–13).

Extravasation is a complication related to the infusion of chemotherapy drugs in peripherally inserted TIVADs because it is clearly connected to the greater mobility of the arm compared to the chest (13).

Extravasation is one of the most feared events related to the chemotherapy drug infusion. Inadvertent administration of a solution or drug into the tissue surrounding the intravenous catheter can, in fact, result in serious complications. In particular, if it is a solution or a non-vesicant drug, it is called infiltration; when it comes to a vesicant drug, it is called extravasation. Both infiltration and extravasation can have serious consequences: the patient may require surgery that causes large scarring, experience functional limitations, or even require amputation (14). Chemotherapy extravasation remains an accidental complication of chemotherapy administration and may result in serious damage to patients (15).

A recent study evaluated a total of 739,812 infusions and identified 673 extravasation events. Incidence for all extravasation events was 0.09% (16).

More specifically, chemotherapy agents may be classified by their potential to cause tissue necrosis. Vesicants are agents that can cause blistering, sloughing of the skin, and varying degrees of localized tissue damage. Non-vesicants do not impair or destroy the tissue when they infiltrate into the tissue (17). Vesicant chemotherapy agents can be divided into two categories: DNA binding and DNA non-binding. Vesicants that bind to nucleic acids in DNA (e.g., anthracyclines) bind to the DNA in the cells of healthy tissue when they extravasate from the vein and promptly cause cell death. DNA-doxorubicin complexes are released from dead cells in the tissue and are taken up by adjacent healthy cells by endocytosis. This process of cellular uptake of extracellular substances sets up a continuing cycle of tissue damage as the anthracycline is retained in the tissue for a long period of time and recirculated in the surrounding area (18).

To prevent serious and permanent damage due to extravasations, early identification has particular importance. Generally, the optimal treatment of anthracycline extravasation includes local tissue cooling, elevation of the afflicted extremity, dexrazoxane administration, and possibly topical DMSO (19).

According to our hospital procedure, dexrazoxano must be used in case of anthracycline extravasation. Dexrazoxano works with two different mechanisms: first, the iron chelation caused by its open-ring metabolite, which can reduce iron-dependent oxidative stress that is responsible for anthracycline cardiotoxicity; second, dexrazoxano causes topoisomerase II inhibition.

The relative contribution of each mechanism to the prevention of tissue damage following anthracycline extravasation remains unclear.

Dexrazoxano must be administered once a day for three consecutive days, according to the following scheme: Day 1: 1,000 mg/m^2^; Day 2: 1,000 mg/m^2^; and Day 3: 500 mg/m^2^. The first infusion must start as soon as possible and, in any case, within the first 6h of the event. Days 2 and 3 treatment should begin at the same time as Day 1 (± 3h). When extravasation involves central venous access, the hospital procedure requires the nurse to block the infusion and aspirate the utmost possible quantity of solution through the catheter.

In this case report, we want to report our experience in treating anthracycline doxorubicin extravasation related to a PICC-port in a patient with breast cancer to provide further evidence of the need for tunneling as protection of the vascular nervous axis of the arm and to save healthy tissue useful in the skin reconstruction phase. This antineoplastic chemotherapeutic agent is known to cause severe and progressive tissue necrosis. Extravasation may also produce pain and/or a burning sensation in the area where doxorubicin was administered intravenously. Doxorubicin extravasation creates a severe tissue necrosis, which is unusual because it may not appear until several weeks later and may continue to worsen for several months (20).

Furthermore, we aim to highlight the importance of a rapid, multidisciplinary intervention, as defined by a hospital-adopted procedure for the management of this specific type of extravasation. In particular, for the PICC-port, this intervention begins with the careful planning of the device placement, including appropriate tunneling.

## 2 Case description

A 63-year-old female patient was, with no comorbidities but overweight, admitted to our hospital in 2023. She had previously undergone a left mastectomy with axillary dissection. The oncologist requested the placement of a long-term central venous access for chemotherapy, expected to last at least 5–6 months.

After a consultation and the patient examination, it was agreed to place a PICC-port. An ultrasound study of the venipuncture site was performed according to the Rapid Peripheral Vein Assessment (RaPeVA) protocol. The venipuncture site was marked with a black dermatographic pen laterally to the ultrasound probe, in the upper proximal area of the arm. At this level, it was generally possible to identify a brachio-axillary vein with a diameter of at least 0.5 cm capable of accommodating a 5 Fr PICC port catheter, which was the PICC port catheter size.

Afterwards, the feasibility of tunneling and creating a subcutaneous pocket in the “green zone” of Dawson’s ZIM system to place the reservoir was evaluated. Given the size of the arm and the subcutaneous tissue, it was decided to perform tunneling parallel to the vascular-nervous bundle for about 7 cm in order to position the reservoir in the median area of the arm on the medial side.

Using maximum barrier protections, aseptic technique, and real-time ultrasound guidance, after local anesthesia with 2% lidocaine, venipuncture was performed with a micro-introduction kit and indirect Seldinger technique. Next, the venous catheter was introduced, and its length was evaluated using tip location and tip navigation systems. This was followed by the creation of the pocket for the reservoir and retrograde tunneling, still under local anesthesia. Finally, after testing the catheter’s functionality, suturing was performed with separate inverted intradermal stitches using a 4–0 absorbable monofilament thread, and adhesive (cyanoacrylate) was applied. The procedure concluded with medium-pressure dressing, and the patient was given an appointment for 4–5 days later, before the first chemotherapy infusion, for reevaluation of the surgical wound and catheter functionality.

**Figure 1:**
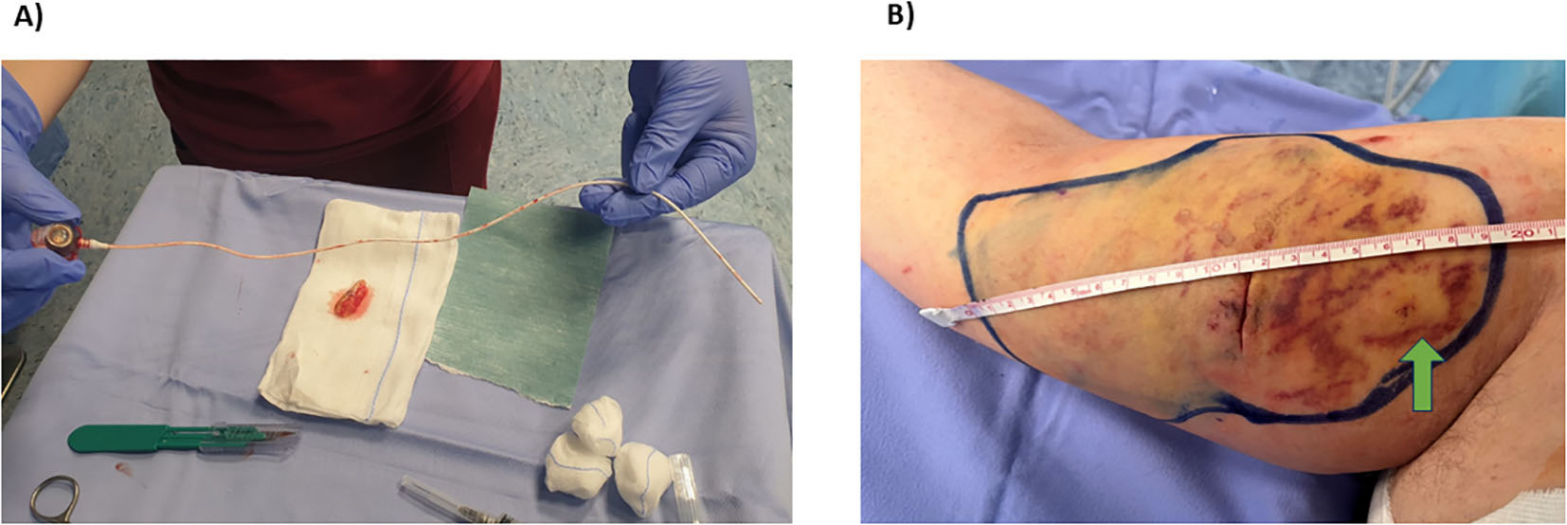
**(A)** Intact PICC-port after removal; **(B)** Edematous and reddened limb post PICC-port removal. Note the diameter of the extravasation spread and the visible venipuncture site, apparently included in the area affected by the extravasation.

The patient presents to the chemotherapy clinic for scheduled treatment with doxorubicin and cyclophosphamide. A Huber needle was placed, and the catheter’s functionality was verified. About an hour after the start of therapy, an alteration in the anatomical profile of the arm with edema and redness was noted. The patient complains of burning, and the Huber needle was found to have dislodged from the reservoir. The institutional procedure for extravasation was started immediately. The needle was removed, and the oncologist, plastic surgeon, and anesthesiologist have been contacted. The patient was taken to the operating room where the PICC-port was removed (**Figure 1A**), a short-term triple-lumen CVC was placed with ultrasound-guided access in the left jugular, and a diffuse extravasation with a diameter of about 20 cm was observed. Apparently, the 7 cm of tunneling was not enough to protect the vascular-nervous bundle of the arm.

The patient was properly medicated and treated with the dexrazoxano (Savene^®^) antidote according to institutional procedure: the extent of the lesion at the venipuncture site was reduced; lesions from vesicant chemotherapy appeared (**Figure 1B**); reduced venous compressibility was noted, antibiotic therapy continued, and thromboembolic prophylaxis therapy (chemical phlebitis) was initiated.

**Figure 2:**
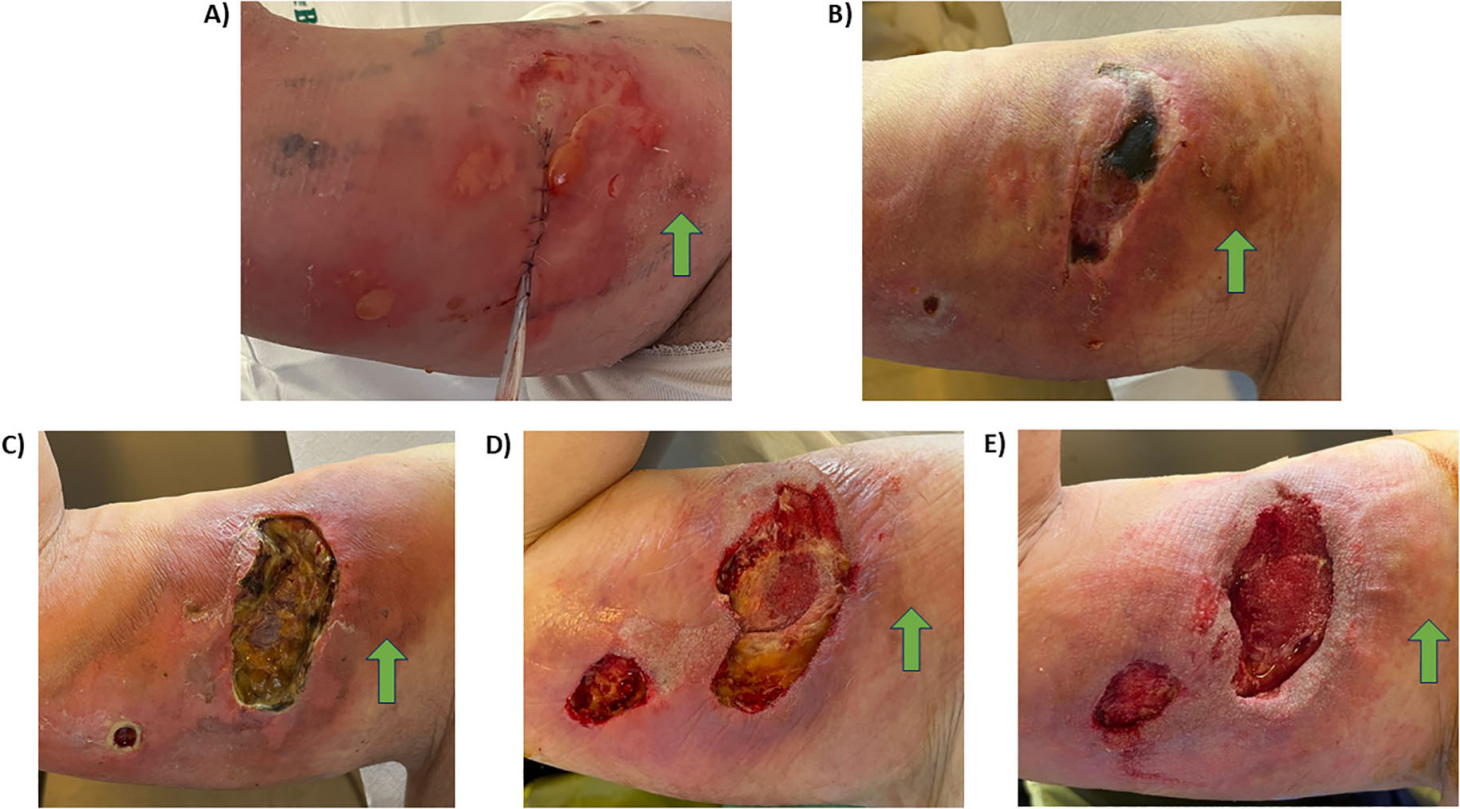
**(A)** Phase 1. About a week later, a reduction in inflammation due to the caustic properties of the vesicant chemotherapy was noted. **(B)** Phase 2. Approximately 1 month later, stabilization of the cutaneous and subcutaneous necrosis was observed. Initially, the dressing included cortisone and antibiotic creams (Clobesol and Gentamicin) and Phytostimoline. **(C)** Phase 3. Two months later, definition of the necrosis with tissue ulceration down to the muscle fascia was observed, exposing the biceps muscle. **(D)** Phase 4. Three months later, surgical cleansing and debridement were performed, followed by application of VAC Therapy for 60 days, interrupted 3 times for dressings with gauze soaked in Betadine due to allergy to the VAC patch. **(E)** Phase 5: Four months later, cleansing was performed with Noruxol and dressings with Phytostimoline in anticipation of scheduling surgical intervention for decontamination and repair with grafts or flaps. The venipuncture site is indicated.

The necrotic area began to demarcate and was removed through surgical debridement; thromboembolic prophylaxis continued (**Figures 2A, B**). A thoracic port was placed on the left side for the continuation of therapy.

The patient continued to receive regular care, including surgical debridement; thromboembolic prophylaxis was ongoing (**Figure 2C**).

Continued dressings and surgical debridement were ongoing; thromboembolic prophylaxis was progressively reduced, and vacuum-assisted closure (VAC) therapy was initiated (**Figures 2D, E**).

**Figure 3:**
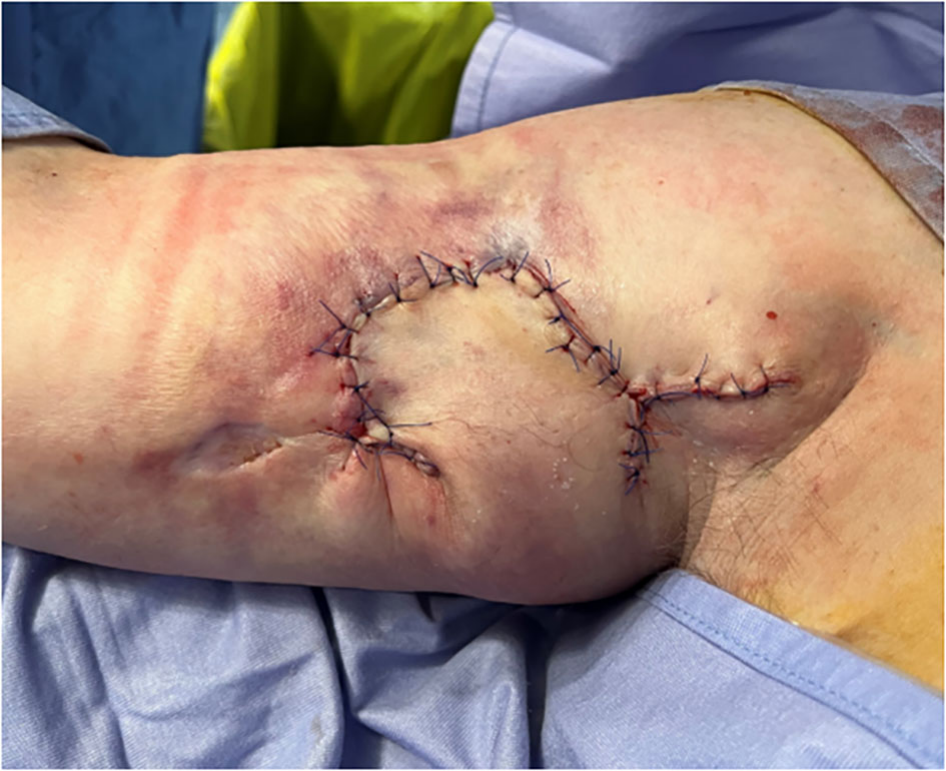
The patient has taken medication regularly. After 10 months, reconstructive plastic surgery was scheduled using a local skin flap based on the tunneling zone, including the indicated venipuncture site.

After 10 months of wound healing among different pathways without any real advantage, we had a wound about 6 cm × 4 cm. The patient had a real discomfort caused by perilesional skin irritation and for continuous liquid secretions. So, in this case, we have planned a plastic surgery procedure with a local skin flap based on a safe vessel apport. This skin flap was obtained from the portion of skin that contained the venipuncture site (**Figure 3**). The same skin flap was saved from the necrotizing action of the extravasated drug due to the presence of tunneling. This provides further evidence of the need for tunneling as protection of the vascular nervous axis of the arm and to save healthy tissue useful in the skin reconstruction phase.

**Figure 4:**
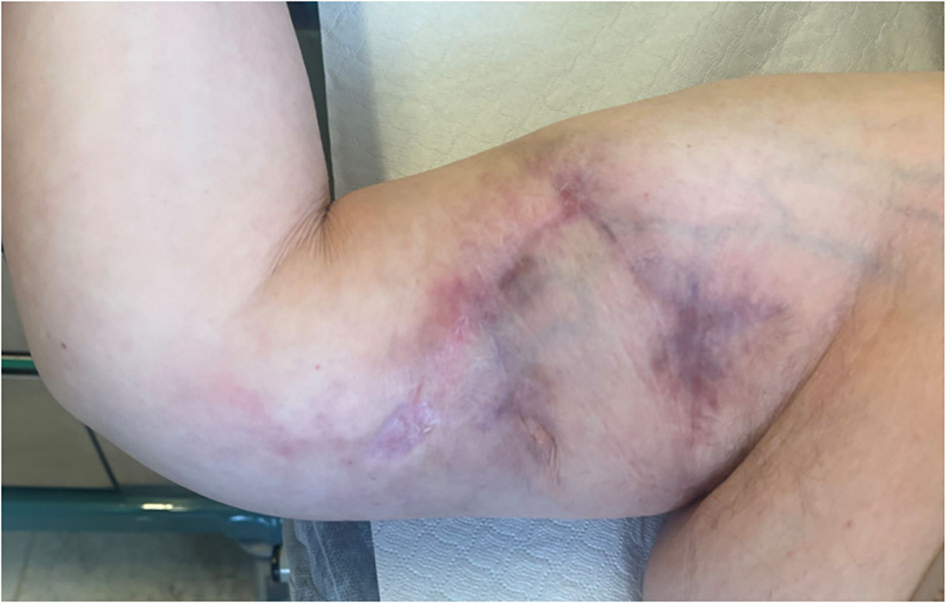
Follow up of the patient at 6 months.

Previously, we performed an escharotomy surgery of the wound, and after that, previous to a skin marking, we made a skin incision until the muscle fascia, and we have rotated a cutaneous and subcutaneous flap. All the environment was full of scars and of cicatricial adherences, probably the result of chemotherapy extravasation. Even if we have found this obstacle, we have been provided with good tissue and good coverage of the wound with our flap (**Figure 3**). After the surgery, we got 15 days for final closure without any complications. We had the patient in follow-up for 6–12 months with optimal results of the scar (**Figure 4**).

## 3 Discussion

Venous access ports positioned in the upper arm are a safe device for administering chemotherapy in breast cancer patients. This type of device is usually well accepted, especially if the positioning is done in a personalized way. In this case, it is often preferred to both the PICC and the chest port (1, 2, 6, 9).

In any case, each type of implantable device must be monitored and managed in relation to the specific placement.

As for the positioning of the PICC, a feasibility study of venipuncture with the support of the ultrasound technique must also be carried out for the PICC port in order to choose a caliber that respects the venous heritage. This evaluation allows us to prevent the thrombotic event, which is particularly related to multiple venipunctures and positioning in small-caliber vessels.

As for the positioning of the chest port, another assessment must be performed to evaluate the better surgery site and the tunnelization, which appears to play a crucial role in the successful management of extravasation, as demonstrated by our clinical experience.

In this specific case report, tunneling played a protective role at the infusion site, preventing the extravasated chemotherapeutic agent from infiltrating deeper tissues and causing extensive damage that could have impaired function or even resulted in limb loss. The skin flap spared from exposure to the drug was later utilized by the plastic surgeon during the reconstructive phase, as shown in **Figure 3**.

It must be considered the reservoir size and the specific arm anatomy. The best choice of the surgery site could prevent error during the introduction of the Huber needle and the possible consequent extravasation. It is easy to understand how the Huber needle is more likely to dislocate during infusion when the reservoir is positioned at the level of the upper limb, which in itself is more mobile than the chest.

The multidisciplinary team should also pay attention to the administration phase with the PICC port. Therefore, when chemotherapy is administered, the patient must be adequately involved and motivated to keep the arm still and to report any pain or burning felt at the infusion site.

A PICC port could favor the necrotizing effect of the extravasated drug, in particular in skinny patients and in the absence of tunnelization because of the injury of the neurovascular bundle. From this perspective, early removal of the device and tunneling are recommended.

Past studies indicated that the overall estimated incidence of chemotherapy extravasation ranges from 0.01% to 7% (21). Other authors report an incidence of chemotherapy extravasation ranging from 0.1% to 6% for peripheral venous access devices and from 0.26% to 4.7% for central venous access devices (15). Data on the incidence is scant due to the absence of a centralized register of chemotherapy extravasation events.

There have been several reports of extravasation with the use of chest ports in breast cancer (22), in Ewing’s sarcoma (23), and in acute lymphoblastic leukemia patients (24), in a pediatric patient receiving paclitaxel, likely for a solid tumor (25), and in a neonate requiring calcium chloride infusion through a central venous port (26).

In another study, 1,320 patients were included, with 794 in the PORT group and 526 in the PICC group. The overall complication rate was significantly lower in the PORT group (*p* = 0.05). Catheter malfunction occurred less frequently in the PORT group compared to the PICC group (*p* < 0.01). Moreover, thrombotic events were significantly less common in the PORT group (*p* = 0.02). No significant differences were observed between the two groups in terms of operative complications, catheter migration, malposition, extravasation, infections, or complications requiring catheter removal (27).

Nevertheless, to the best of our knowledge, this is the first report of a documented doxorubicin extravasation from a PICC-port in a patient with breast cancer.

The positioning technique of the arm port or PICC port changes the degree of safety in the use of the device. As well as reducing the incidence of catheter-related complications such as thrombosis and infection (1), the tunneling increases the chances of protection of the arm nerve bundle in case of overflow. In order to fulfill safe tunneling, it would be necessary to perform a feasibility assessment of the reservoir pocket prior to making the sterile field and after locating the venipuncture site using the real-time ultrasound guidance. In fact, the venipuncture site, tunnel placement, and pocket realization site should also be well identified to carry out the sterile field for performing the placement.

As placement and management of arm ports and PICC ports require the activation of a multidisciplinary team, so too does the management of extravasation complications require prompt multidisciplinary intervention. In most hospitals where chemotherapy is administered, the use of central venous catheters is now widespread to try to limit extravasation as much as possible.

The Italian Ministry of Health published the Raccomandazione 14 on the prevention of errors in treatments with antineoplastic drugs (28). In Raccomandazione 14, section 4.6.e., correct manipulation of venous access, it is recommended that for patients who have to perform a program of periodic infusions of antineoplastic drugs, implantation of central and peripheral venous catheters is considered useful to reduce the risk of extravasation. Shared procedures should be adopted among the operating units involved for the insertion of the medical device, and considering the relevance for the prevention of healthcare-related infections, it is essential to ensure proper management of venous access at all times. In any case, these medical devices are not sufficient to avoid the danger, so much so that Raccomandazione 14 itself calls for the preparation of a specific and updated procedure for the management of extravasation. This document should be immediately accessible to the health professionals involved and should indicate the first intervention measures. Therefore, it is essential to carry out training of all involved operators and to create a dedicated kit with identified antidotes for each type of chemotherapy drug. This kit should include at least cannula needles and needles of different calibers, water for injectable preparations, 10 ml vials of 25% sodium thiosulfate, hyaluronidase, vials of 99% dimethyl sulfoxide (DMSO), 1% hydrocortisone cream, sterile gauze and sterile syringes, hot and cold pack systems, and a black dermographic marker. It should be remembered that local heat treatments are used to reduce the local reaction and absorption of the infiltrate. Cooling the site (with ice packs) facilitates vasoconstriction, theoretically limiting drug dispersion.

In addition to specific antidotes, some general measures are recommended, including immediately stopping the infusion, taking care to leave the cannula in place. This will, in fact, make it possible to aspirate as much of the extravasated drug as possible. If extravasation has involved a limb, it is advisable to elevate and immobilize it. In some cases, it may be necessary to consult a surgeon.

It would be recommended for every hospital managing oncology patients to produce local protocols that facilitate the treatment of extravasation when necessary. Collaboration and proper information for the patient and caregiver would facilitate the reduction of the magnitude of the complication of extravasation because it would allow faster secondary prophylaxis.

## 4 Patient perspective

Following the surgical and pharmacological treatments described above, the patient regained full limb functionality and was able to resume all regular activities. This favorable outcome was primarily due to the prompt and skilled intervention of the multidisciplinary team, which effectively minimized the serious complications typically associated with doxorubicin extravasation. Since the incident occurred during the first cycle of adjuvant therapy with doxorubicin and cyclophosphamide, the patient was deemed ineligible to continue with anthracycline. As a result, the treatment plan was modified, and she proceeded with the trastuzumab/Paclitaxel regimen for 12 cycles, followed by 6 additional cycles of trastuzumab monotherapy, completing a total of 18 administrations. The entire course of therapy was successfully completed.

## Data Availability

The original contributions presented in the study are included in the article/supplementary material. Further inquiries can be directed to the corresponding author.
